# Yellow fever virus infection in non-human primates: a systematic review and meta-analysis of prevalence, seroprevalence, and epizootic case reports (1950–2025)

**DOI:** 10.3389/fvets.2026.1798623

**Published:** 2026-03-31

**Authors:** D. Katterine Bonilla-Aldana, Jorge Luis Bonilla-Aldana, Dayana M. Calle-Hernández, Jaime E. Castellanos, Lysien Zambrano, Alfonso J. Rodriguez-Morales

**Affiliations:** 1 College of Medicine, Korea University, Seoul, Republic of Korea; 2 Grupo de Virologia, Universidad El Bosque, Bogotá, Colombia; 3 Grupo Colaborativo de Investigación en Enfermedades Transmitidas por Vectores, Zoonóticas y Tropicales de Risaralda (GETZ), Pereira, Colombia; 4 Facultad de Odontología, Universidad Nacional de Colombia, Bogotá, Colombia; 5 Department of Morphological Sciences, School of Medical Sciences, Universidad Nacional Autónoma de Honduras, Tegucigalpa, Honduras; 6 Faculty of Health Sciences, Universidad Científica del Sur, Lima, Peru; 7 Grupo de Investigación Biomedicina, Faculty of Medicine, Fundación Universitaria Autónoma de las Américas-Institución Universitaria Visión de las Américas, Pereira, Colombia

**Keywords:** epizootics, non-human primates, One Health, prevalence, seroprevalence, systematic review and meta-analysis, yellow fever

## Abstract

**Introduction:**

Yellow fever remains a major mosquito-borne viral disease of global public health and ecological concern. Non-human primates (NHPs) are central to the sylvatic transmission cycle and serve as key sentinels of viral circulation. Yet, evidence on yellow fever virus (YFV) infection in NHPs is dispersed and has not been synthesized comprehensively.

**Objective:**

To systematically review and meta-analyze global data on the prevalence and seroprevalence of YFV infection in NHP, and to summarize molecular, clinical, and pathological findings from reported epizootic cases.

**Methods:**

We conducted a systematic search of Scopus, PubMed, Web of Science, and SciELO for studies published between 1950 and 2025, following PRISMA guidelines. Observational studies reporting YFV prevalence or seroprevalence in NHPs were included in the quantitative syntheses, and individual case reports were analyzed separately. Random-effects meta-analyses were performed, with subgroup analyses by geographic region, diagnostic method, and primate genus.

**Results:**

Thirty-nine articles assessing 7,183 NHP met the inclusion criteria; 28 contributed to meta-analyses, and 10 provided 19 individual case reports. Pooled molecular prevalence by RT-PCR was 30.7%, and prevalence by immunohistochemistry was 43.4%, both with substantial between-study heterogeneity. Seroprevalence estimates ranged from 5.0 to 36.4% across assays and settings. Higher infection metrics were observed in howler monkeys and titi monkeys. All reported individual cases were fatal and predominantly associated with severe hepatic and multisystemic pathology. Most data originated from the Americas, particularly Brazil, with limited representation from African endemic regions.

**Conclusion:**

YFV infection in NHP is widespread, often severe, and epidemiologically significant. Our findings underscore the critical sentinel role of NHPs and highlight the need to strengthen integrated One Health surveillance systems to inform prevention and control strategies, particularly in the context of the current resurgence of yellow fever in Latin America and persistent data gaps in Africa.

## Introduction

Yellow fever (YF) remains one of the most critical mosquito-borne viral hemorrhagic diseases affecting humans and animals, despite the long-standing availability of a safe and effective vaccine (1–5). The yellow fever virus (YFV) is maintained through complex transmission cycles involving mosquito vectors and vertebrate hosts, and its epidemiology continues to evolve in response to demographic, environmental, and social change (6–8). In Africa, where YFV is historically endemic, recurrent outbreaks persist in rural and urban areas, often driven by heterogeneous population immunity, rapid urbanization, and limitations in sustained surveillance and vaccination coverage (3, 8, 9). In the Americas, YF is primarily maintained through sylvatic transmission cycles; however, the potential for spillover into peri-urban or urban settings remains a constant concern wherever competent vectors and susceptible human populations coexist (4, 10–13).

In recent years, particularly during 2024 and 2025, South America has experienced a notable resurgence of yellow fever activity, with increasing numbers of confirmed human cases (14), high case-fatality rates (15), and widespread epizootics in NHPs (6, 12, 16–18). These events have affected multiple countries and ecological regions, including areas previously considered low- or moderate-risk. The re-emergence and expansion of YF in such settings underscores persistent vulnerabilities in prevention and control strategies, especially in regions with suboptimal vaccination coverage and expanding human–wildlife interfaces (2–4, 10, 19, 20).

NHPs play a central role in the sylvatic transmission cycle of YFV in the Americas, acting as amplifying hosts and highly sensitive sentinels of viral circulation (21). Epizootics in NHP populations often precede or coincide with human cases and have long been recognized as critical early warning signals of impending outbreaks (1, 16, 19, 21). Despite their epidemiological importance, the literature on YFV infection in NHPs remains limited and fragmented (1, 16, 19, 21). Most studies focus on localized outbreaks, specific regions, or a narrow range of species, particularly highly susceptible genera, such as howler monkeys. Consequently, current evidence provides an incomplete picture of broader infection patterns across different primate taxa, geographic regions, and ecological contexts (21).

Emerging reports of fatal YFV infections in captive and managed-care NHPs, spanning multiple genera and including threatened and endangered species, further highlight existing knowledge gaps (16, 19). These observations indicate that YFV exposure is not confined to free-ranging populations and that zoological collections, wildlife rescue centers, and conservation areas may also be at risk of sylvatic transmission. Beyond their implications for animal health and biodiversity conservation, such events raise important public health concerns by signaling active viral circulation in environments where human exposure is possible (22).

The recent resurgence of yellow fever must be interpreted within a One Health framework that recognizes the interconnectedness of human, animal, and environmental health. Deforestation (23), habitat fragmentation, agricultural expansion, climate variability, and changing vector ecology all contribute to altered transmission dynamics. Social determinants, such as population mobility, occupational exposure in forested areas, informal settlements at forest edges, and inequities in access to vaccination, further exacerbate vulnerability and complicate outbreak detection and response. In this context, robust and synthesized evidence on YFV infection in NHPs is essential to guide integrated surveillance, refine risk assessment, and support timely public health and veterinary decision-making (24, 25).

To our knowledge, no previous systematic review with meta-analysis has comprehensively synthesized the global evidence on YFV infection in NHPs, integrating prevalence, seroprevalence, and epizootic case reports. The present study addresses this gap by systematically reviewing the literature from 1950 to 2025, quantifying infection metrics across regions and primate taxa, and collating individual case-level clinical and pathological data. By doing so, this review aims to strengthen the evidence base for epizootic surveillance and inform One Health strategies for the prevention and control of yellow fever in both animals and human populations.

## Methods

**Figure 1:**
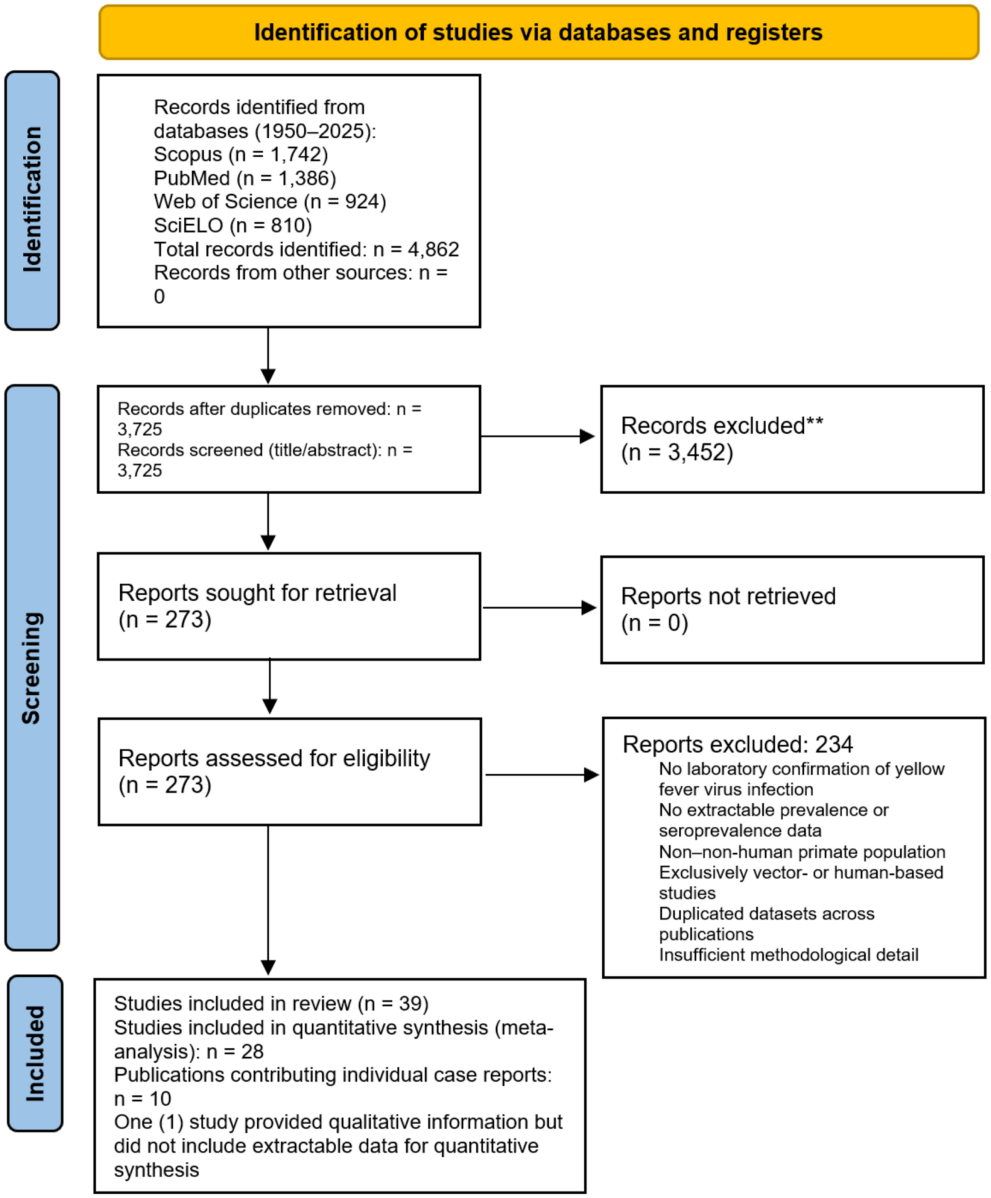
PRISMA diagram flow.

This study was conducted and reported in accordance with the Preferred Reporting Items for Systematic Reviews and Meta-Analysis (PRISMA) guidelines (Figure 1) (26).

### Information sources and search strategy

We performed a comprehensive bibliographic search to identify studies evaluating the prevalence or seroprevalence of YFV infection in NHPs (Supplementary Table S1). Four electronic databases were queried: Scopus, Web of Science, PubMed, and SciELO. The search strategy was developed and peer-reviewed using the PRESS (Peer Review of Electronic Search Strategies) checklist to optimize sensitivity and specificity. No language restrictions were applied. The search covered all records from database inception to 1st December 2025.

### Study selection and data extraction

We included observational studies reporting the prevalence or seroprevalence of YFV infection in NHPs, based on laboratory confirmation. Systematic, scoping, and narrative reviews, editorials, and letters to the editor were excluded. Case reports and small case series were not incorporated into the meta-analyses but were retained for a separate qualitative synthesis of molecular, clinical, and pathological features.

All records identified through the search strategy were imported into the Rayyan QCRI data management software (27). Duplicate articles were identified and removed. Title and abstract screening was then performed, followed by full-text assessment of potentially eligible articles. Studies that did not meet the predefined inclusion criteria were excluded. All screening steps were conducted independently by four reviewers. Disagreements were resolved by discussion and consensus among all authors. Data from included articles were extracted using a standardized template created in Microsoft® Excel®. The following variables were collected: first author, year of publication, country, study period, diagnostic method, type of study population, primate taxa, sample size, and number of NHPs with evidence of YFV infection or past exposure.

### Risk of bias assessment

The risk of bias was assessed using the Newcastle–Ottawa Scale (NOS) adapted for cross-sectional and observational studies (28). The NOS was selected because most included investigations employed observational designs typical of wildlife and outbreak surveillance, for which randomized or controlled frameworks are generally not feasible. The scale enables structured evaluation of key methodological domains, including the selection of the study population, the comparability of study groups, and the ascertainment of outcomes. Given that many studies relied on epizootic surveillance of dead or clinically affected animals and frequently used non-probabilistic sampling strategies, the NOS provided a standardized framework to assess potential biases in representativeness, diagnostic ascertainment, and completeness of reporting. Studies with NOS scores ≥7 stars were classified as having low risk of bias, and those with scores ≤6 stars were classified as high risk of bias. Two reviewers independently performed the risk bias assessments; discrepancies were resolved by consensus. Detailed scores for each domain are presented in Supplementary Table S2.

### Publication bias assessment

Formal assessment of publication bias was not undertaken. Recent methodological work indicates that conventional tools such as funnel plots and Egger’s test are unreliable and potentially misleading for proportional meta-analyses of prevalence data. These approaches were developed under the assumption that studies with “positive” or statistically significant findings are more likely to be published; however, this assumption is difficult to operationalize for prevalence outcomes, for which no clear definition of a “positive” result exists. Moreover, there is no strong evidence that suggests that proportions conform to the distributional assumptions underlying these tests. In light of these limitations, and consistent with current recommendations, we did not apply funnel plots or regression-based tests for small-study effects (29, 30).

### Statistical analysis

Quantitative syntheses were performed using the statistical analysis program Stata v. 16®, JASP, and OpenMetanalyst. Prevalence and seroprevalence estimates were pooled using a random-effects model (Dersimonian and Laird) to account for anticipated between-study heterogeneity. Exact 95% confidence intervals were calculated using the Clopper-Pearson method. To stabilize the variance of proportions, we applied the Freeman-Tukey Double arcsine transformation before pooling. Between-study heterogeneity was assessed using Cochran’s *Q* test and the *I*^2^ statistic. *I*^2^ values >60% were interpreted as indicating substantial heterogeneity, and a *Q*-test *p*-value < 0.05 was considered evidence of statistically significant heterogeneity. Pre-specified subgroup analyses were conducted by diagnostic assay, world region, and by NHP genus. We performed a sensitivity analysis excluding studies at high risk of bias to evaluate the robustness of pooled estimates.

## Results

### Selection of studies

The systematic search of Scopus, PubMed, Web of Science, and SciELO databases for the period 1950–2025 identified 4,862 records. After removal of 1,137 duplicates, 3,725 titles and abstracts were screened. Of these, 3,452 records were excluded as irrelevant or not meeting the inclusion criteria. A total of 273 full-text articles were assessed for eligibility; 234 were excluded, primarily because they lacked laboratory confirmation of YFV infection, did not report extractable prevalence or seroprevalence data, contained duplicated datasets, or focused on non–NHPs populations. Ultimately, 39 articles met the eligibility criteria and were included in the systematic review. Of these, 28 studies contributed data to the quantitative meta-analysis (18, 24, 31–57), 10 were individual case reports or small case series analyzed separately (16, 58–66), encompassing 19 NHP cases, and 1 study provided qualitative information but lacked extractable quantitative data. The study selection process is summarized in the PRISMA flow diagram (Figure 1).

### Characteristics of included studies

Key characteristics of the included articles are summarized in Table 1. Overall, 39 studies evaluating 7,183 NHPs were included. The publication period spanned 1952 to 2024; the majority of data were published after 2016, with a notable peak in 2020 (28.2%) (Table 1). Of the total NHPs assessed, 87% originated from the Americas and 13% from Africa (Table 1). No eligible studies were identified from other regions. Brazil was the most represented country, contributing 73% of all NHP data (Table 1). Regarding diagnostic methods, RT-PCR was used in 6 studies involving 6,649 NHPs, and genome sequencing was performed in 6 studies covering 1,109 NHPs. Immunohistochemistry was applied to 7,183 NHPs. Serological assays included hemagglutination inhibition (346 NHPs), plaque reduction neutralization test (PRNT, 646 NHPs), mouse protection tests (447 NHPs), and ELISA (1 NHP).

**Table 1:** Studies included assessing YF in NHP.

Title study	Publication year	Study years	Continent	Country	Study type	Species	Alive/dead	N	n	Samples
RT-PCR										
Yellow fever: reemerging in the state of São Paulo, Brazil, 2009	2013	2009	Americas	Brazil	Prevalence	*Allouatta* sp.	Dead	51	2	Tissue
							Dead	5	0	Tissue
Yellow fever epizootics in non-human primates, Southeast and Northeast Brazil (2017 and 2018)	2020	2018	Americas	Brazil	Prevalence	*Allouatta* sp.	Dead	76	43	Liver, kidney, brain tissue and whole blood
						*Callithrix* spp.	Dead	1,505	140	Liver, kidney, brain tissue and whole blood
						*Sapajus* spp.	Dead	38	8	Liver, kidney, brain tissue and whole blood
						*Callicebus* spp.	Dead	5	5	Liver, kidney, brain tissue and whole blood
						*Leontopithecus* spp.	Dead	24	1	Liver, kidney, brain tissue and whole blood
						Unknown	Dead	448	62	Liver, kidney, brain tissue and whole blood
Yellow fever epizootics in non-human primates, São Paulo state, Brazil, 2008–2009 *^,^**	2013	2008–2009	Americas	Brazil	Prevalence	Unknown	Dead	66	2	Liver, spleen, kidney, heart, lung, brain and blood
Possible non-sylvatic transmission of yellow fever between non-human primates in São Paulo city, Brazil, 2017–2018 **	2020	2017–2018	Americas	Brazil	Prevalence	*Alouatta guariba clamitans*	Dead	209	140	Liver, brain and spleen
						*Callithrix* sp.	Dead	324	22	Liver, brain and spleen
						*Sapajus nigritus*	Dead	7	0	Liver, brain and spleen
						*Callicebus nigrifons*	Dead	1	0	Liver, brain and spleen
YELLOW ALERT: Persistent Yellow Fever Virus Circulation among Non-Human Primates in Urban Areas of Minas Gerais State, Brazil (2021–2023)	2024	2021–2023	Americas	Brazil	Prevalence	*Callithrix* spp.	Dead	148	16	Liver and lung
						*Alouatta* sp.	Dead	4	0	Liver and lung
							Dead	14	2	Liver and lung
Ecological drivers of sustained enzootic yellow fever virus transmission in Brazil, 2017–2021	2023	2017–2021	Americas	Brazil	Prevalence	*Callithrix* spp.	Dead	175	0	Liver
						*Alouatta* spp.	Dead	2	0	Liver
							Dead	10	0	Liver
							Dead	44	1	Lung
Detection of antibodies against Icoaraci, Ilhéus, and Saint Louis Encephalitis arboviruses during yellow fever monitoring surveillance in non-human primates (*Alouatta caraya*) in southern Brazil ***^,^****	2019	2015–2016	Americas	Brazil	Prevalence	*Alouatta caraya*	Live	26	0	Serum
Yellow Fever Outbreak Affecting Alouatta Populations in Southern Brazil (Rio Grande do Sul State), 2008–2009 *^,^**^,^*****	2010	2008–2009	Americas	Brazil	Prevalence	*Alouatta guariba clamitans*	Dead	218	137	Liver, lung, heart, kidney and spleen
						*Alouatta caraya*	Dead	44	36	Liver, lung, heart, kidney and spleen
Yellow Fever as Cause of Death of Titi Monkeys (*Callicebus* spp.) **	2010	2008	Americas	Brazil	Prevalence	*Callicebus* spp.	Dead	43	18	Liver, kidney, spleen, heart, brain and lung
Serological evidence for potential yellow fever virus infection in non-human primates, southeastern Mexico ****^,^*****	2024	2012–2018	Americas	Mexico	Prevalence	*Ateles geoffroyi*	Live	25	0	Serum
						*Alouatta pigra*	Live	3	0	Serum
						*Alouatta palliata*	Live	2	0	Serum
Outbreak of Yellow Fever among Nonhuman Primates, Espirito Santo, Brazil, 2017 **	2017	2017	Americas	Brazil	Prevalence	*Allouatta* sp.	Live	2	2	Liver, kidney, spleen
						Unknown	Live	20	19	Liver, kidney, spleen
Histopathologic Patterns and Susceptibility of Neotropical Primates Naturally Infected With Yellow Fever Virus **	2017	2008	Americas	Brazil	Prevalence	*Alouatta* spp.	Dead	48	19	Liver, spleen, heart and brain
						*Callithrix* spp.	Dead	1,219	31	Liver, spleen, heart and brain
						*Sapajus* spp.	Dead	26	1	Liver, spleen, heart and brain
						*Callicebus* spp.	Dead	4	2	Liver, spleen, heart and brain
						*Leontopithecus* spp.	Dead	7	1	Liver, spleen, heart and brain
Detection of the mosquito-borne flaviviruses, West Nile, Dengue, Saint Louis Encephalitis, Ilheus, Bussuquara, and Yellow Fever in free-ranging black howlers (*Alouatta caraya*) of Northeastern Argentina ****	2017	2010	Americas	Argentina	Prevalence	*Alouatta caraya*	Live	108	0	Serum
Detección por reacción en cadena de la polimerasa de transcriptasa inversa del virus de la fiebre amarilla en monos silvestres: una herramienta sensible para la vigilancia epidemiológica **	2007	2003–2004	Americas	Colombia	Prevalence	*Alouatta seniculus*	Dead	5	3	Liver
Epidemiologic profile and histopathological findings in Neotropical Primates during and after the yellow fever outbreak in Espírito Santo, Brazil	2022	2017–2020	Americas	Brazil	Prevalence	Multiple genera	Dead	487	160	Liver
Epizootics due to Yellow Fever Virus in São Paulo State, Brazil: viral dissemination to new areas (2016–2017) **	2019	2016–2017	Americas	Brazil	Prevalence	*Alouatta* spp.	Dead	138	114	Liver and brain
						*Callithrix* spp.	Dead	23	23	Liver and brain
						*Sapajus* spp.	Dead	1	1	Liver and brain
Evaluation of arboviruses of public health interest in free-living non-human primates (*Alouatta* spp., *Callithrix* spp., *Sapajus* spp.) in Brazil	2015	2012–2013	Americas	Brazil	Prevalence	*Alouatta* spp.	Live	7	0	Serum
						*Callithrix* spp.	Live	29	0	Serum
						*Sapajus* spp.	Live	44	0	Serum
Neighbor danger: Yellow fever virus epizootics in urban and urban–rural transition areas of Minas Gerais state, during 2017–2018 yellow fever outbreaks in Brazil *	2020	2017–2018	Americas	Brazil	Prevalence	*Alouatta caraya*	Dead	2	1	Liver
						*Alouatta guariba*	Dead	17	12	Liver
						*Alouatta* sp.	Dead	33	28	Liver
						*Callicebus nigrifrons*	Dead	19	12	Liver
						*Callicebus personatus*	Dead	7	6	Liver
						*Callicebus* sp.	Dead	6	4	Liver
						*Callithrix aurita*	Dead	2	1	Liver
						*Callithrix geoffroyi*	Dead	21	10	Liver
						*Callithrix penicillata*	Dead	170	44	Liver
						*Callithrix* sp.	Dead	466	155	Liver
						*Sapajus nigritus*	Dead	1	1	Liver
						*Sapajus* sp.	Dead	1	0	Liver
						Unknown	Dead	36	24	Liver
Survey on Non-Human Primates and Mosquitoes Does not Provide Evidences of Spillover/Spillback between the Urban and Sylvatic Cycles of Yellow Fever and Zika Viruses Following Severe Outbreaks in Southeast Brazil ****	2020	2015–2018	Americas	Brazil	Prevalence	Multiple genera	Live	144	14	Serum
O impacto do vírus da Febre Amarela na população de primatas não humanos da região sul do Município de Ribeirão Preto – SP (2019) ****	2019	2018	Americas	Brazil	Prevalence	*Callithrix penicillata*	Live	38	0	Serum
						*Sapajus nigritus*	Live	1	0	Serum
Immunohistochemistry										
Frequency of histopathological changes in Howler monkeys (Alouatta sp.) naturally infected with yellow fever virus in Brazil	2016	2007–2009	Americas	Brazil	Prevalence	*Allouatta*	Dead	4,339	474	Liver
Hemagglutination inhibition										
Wild terrestrial rainforest mammals as potential reservoirs for flaviviruses (yellow fever, dengue2 and St Louis encephalitis viruses) in French Guiana	2004	1994–1995	Americas	French Guiana	Seroprevalence	*Alouatta seniculus*	Live	98	18	Serum
						*Pithecia pithecia*	Live	5	5	Serum
						*Saguinus midas*	Live	42	2	Serum
Seroepidemiological monitoring in sentinel animals and vectors as part of arbovirus surveillance in the state of Mato Grosso do Sul, Brazil	2012	2010	Americas	Brazil	Seroprevalence	*Alouatta caraya*	Live	2	0	Serum
						*Callicebus donacophilus*	Live	1	0	Serum
						*Sapajus apella*	Live	62	0	Serum
Surveillance of Arboviruses in Primates and Sloths in the Atlantic Forest, Bahia, Brazil	2018	2006–2014	Americas	Brazil	Seroprevalence	*Leontopithecus chrysomelas,*		103	2	Serum
						*Sapajus xanthosternos*		7	2	Serum
PRNT										
Prevalence of antibodies to alphaviruses and flaviviruses in free-ranging game animals and nonhuman primates in the greater Congo basin	2013	2001–2009	Africa	Congo	Seroprevalence	*Gorilla beringei beringei*	Live	32	0	Serum
						*Cercopithecus lhoesti*	Live	5	0	Serum
						*Cercopithecus kandti*	Live	2	0	Serum
						*Pan troglodytes*	Live	2	0	Serum
						*Cercopithecus lhoesti*	Live	3	0	Serum
Seroprevalence of selected flaviviruses in free-living and captive capuchin monkeys in the state of Pernambuco, Brazil	2018	2015–2016	Americas	Brazil	Seroprevalence	*Sapajus* spp.	Live	49	1	Serum
Serosurvey of Nonhuman Primates in Costa Rica at the Human-Wildlife Interface Reveals High Exposure to Flaviviruses	2021	2000–2015	Americas	Costa Rica	Seroprevalence	*Alouatta palliata*	Live	68	0	Serum
						*Cebus imitator*	Live	1	0	Serum
						*Saimiri oerstedii*	Live	3	0	Serum
						*Ateles geoffroyi*	Live	14	0	Serum
Surveillance of Arboviruses in Primates and Sloths in the Atlantic Forest, Bahia, Brazil	2018	2006–2014	Americas	Brazil	Seroprevalence	*Leontopithecus chrysomelas,*	Live	103	2	Serum
						*Sapajus xanthosternos*	Live	7	2	Serum
Mouse protection test										
A Review of the Results of Yellow Fever Protection- Tests on the Sera of Primates from Kenya	1952	1950	Africa	Kenya	Seroprevalence	*Galago* spp.	Live	103	14	Serum
						*Cercopithecus* spp.	Live	261	3	Serum
						*Erythrocebus* spp.	Live	1	0	Serum
						*Papio* spp.	Live	9	0	Serum
						*Colobus* spp.	Live	62	2	Serum
						Unknown	Live	11	0	Serum
ELISA										
Circulation of antibodies against yellow fever virus in a simian population in the area of Porto Primavera Hydroelectric Plant, São Paulo, Brazil	2010	2000–2001	Americas	Brazil	Seroprevalence	*Alouatta caraya*	Live	570	0	Serum

* Also performed genome sequencing. ** Also performed immunohistochemistry. *** Also performed hemagglutination inhibition. **** Also performed plaque reduction neutralization test. ***** Also performed indirect immunofluorescence.

### Risk of bias assessment

Most studies were classified as having a high risk of bias according to the Newcastle–Ottawa Scale. The domains most frequently responsible for lower scores were selection and the representativeness of the study population. Many investigations were embedded within outbreak-driven surveillance systems and focused on dead or clinically affected animals, resulting in non-probabilistic sampling frames and limited representativeness of the broader NHP population. Additional limitations included incomplete reporting of denominators and sampling frames, insufficient description of population characteristics, sampling strategies, and limited control or adjustment for potential confounding variables. In contrast, studies with lower risk-of-bias classifications clearly defined their sampling populations, provided transparent reporting of sample sizes and denominators, and documented laboratory confirmation using validated diagnostic methods such as RT-PCR or immunohistochemistry. These methodological contrasts reflect the operational constraints of epizootic surveillance but should be taken into account when interpreting pooled prevalence and seroprevalence estimates (Supplementary Table S2).

### Prevalence of YFV infection in NHPs by RT-PCR

**Figure 2:**
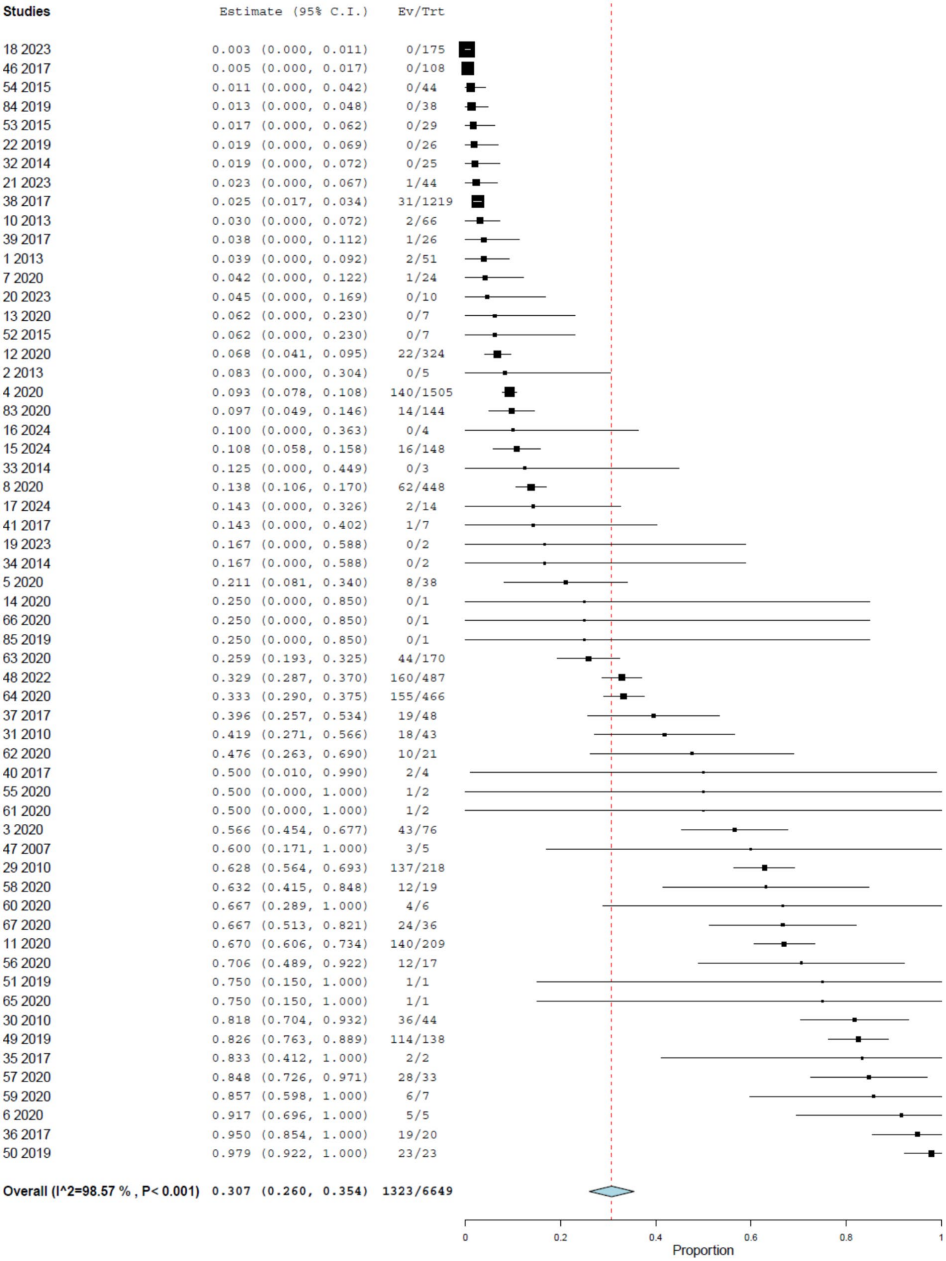
Prevalence of YF infection in NHPs by RT-PCR.

**Figure 3:**
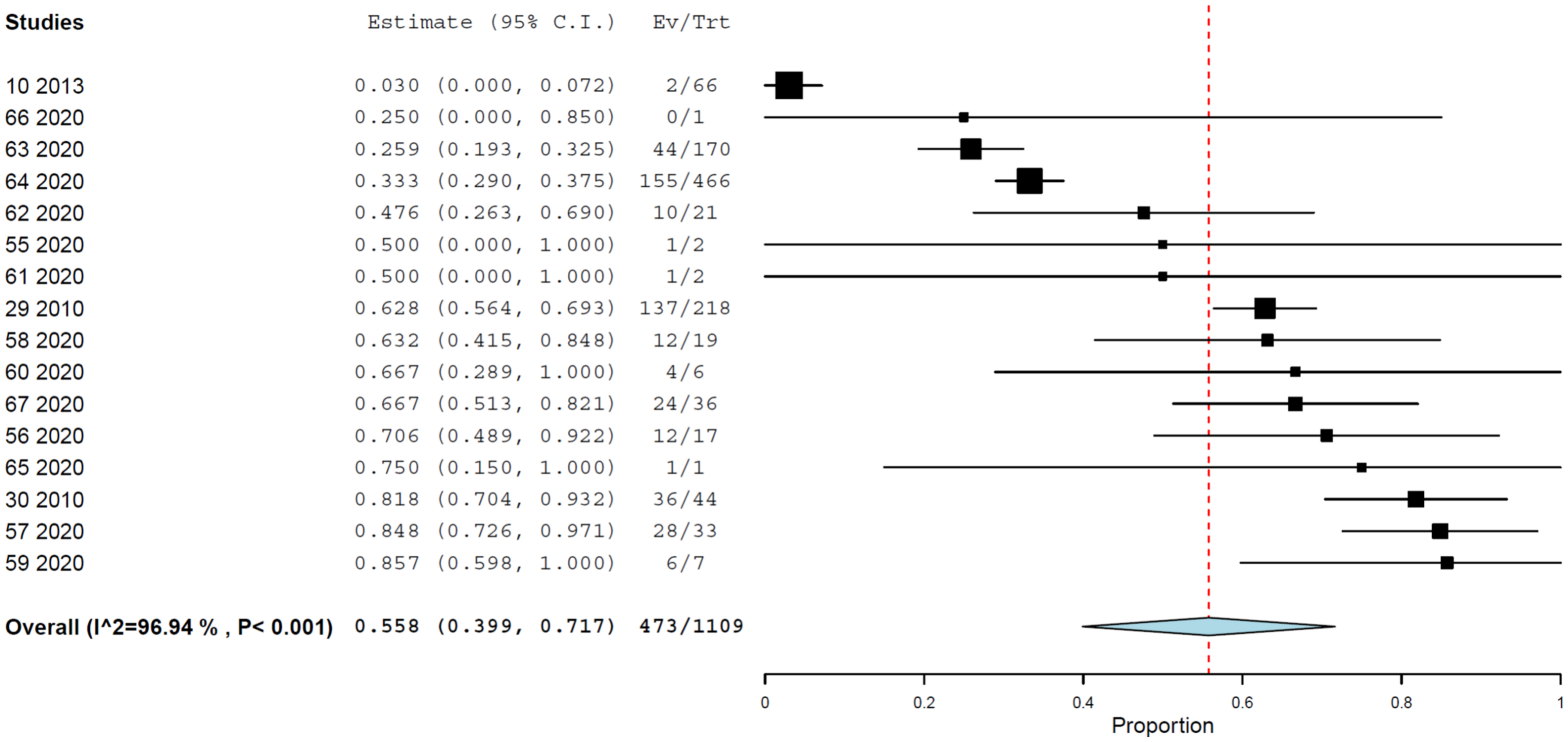
Prevalence of YF infection in NHPs by genome sequencing.

Across all RT-PCR-based studies, the pooled prevalence of YFV infection in NHP was 30.7% (95% CI, 26.0–35.4%), with pronounced between-study heterogeneity (*I*^2^ = 98.566%, τ^2^ = 0.025, *Q*^2^ = 4,045.055) (Figure 2). In a subset of three studies including 1,109 NHPs, YFV infection was additionally confirmed by genome sequencing (Table 1). In this subset, the pooled prevalence of 55.8% (95% CI, 39.9–71.7%), again with high heterogeneity (*I*^2^ = 96.935%, τ^2^ = 0.083, *Q*^2^ = 489.399) (Figure 3).

### Temporal patterns in RT-PCR prevalence

When stratified by period, RT-PCR prevalence estimates were slightly lower in studies conducted before 2020 (29.9%; 95% CI, 21.3–38.5%) than in those conducted from 2020 onwards (31.8%; 95% CI, 25.6–38.1%). Although this difference is modest, it is consistent with intensified epizootic activity and enhanced surveillance in recent years.

### RT-PCR prevalence by NHP genus

**Figure 4:**
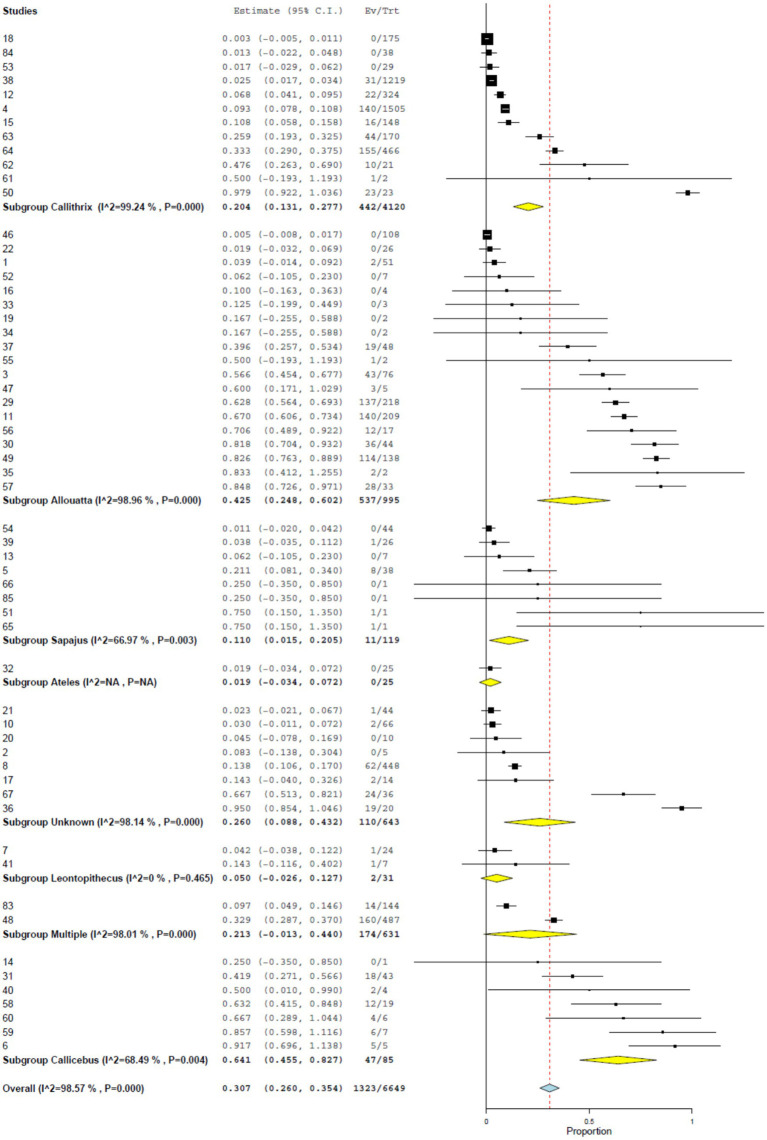
Prevalence of YF infection in NHPs by RT-PCR by species.

Subgroup analyses by genus revealed marked taxonomic differences. Using RT-PCR data, titi monkeys (*Callicebus* spp.) and howler monkeys (*Allouatta* spp.) exhibited significantly higher prevalences than other genera, with a pooled estimate of 64.1% (95% CI, 45.5–82.7%) and 42.5% (95% CI, 24.8–60.2%), respectively (Figure 4 and Table 2). These patterns were confirmed in the subset of NHPs with genome sequencing, where *Callicebus* and *Alouatta* showed prevalences of 71.5% (95% CI, 56.3–86.7%) and 74.1% (95% CI, 61.8–86.3%), respectively, compared with lower estimates in *Sapajus* (50.0, 95% CI, 1.0–99.0%) and *Callithrix* (31.8, 95% CI, 24.8–38.8%).

**Table 2:** Prevalence of YF infection in NHPs by RT-PCR by genus.

Genus	*n*	Estimate	Lower CI	Upper CI	SE	*p*	Z
*Callicebus*	7	64.1	45.5	82.7	0.095	<0.001	6.742
*Allouatta*	19	42.5	24.8	60.2	0.09	<0.001	4.708
*Callithrix*	12	20.4	13.1	27.7	0.037	<0.001	5.484
*Sapajus*	8	11.0	1.5	20.5	0.048	0.023	2.28
*Leontopithecus*	2	5.0	0.0	12.7	0.039	0.196	1.294
*Ateles*	1	1.9	0.0	7.2	0.027	N/A	N/A
Unknown	8	26	8.8	43.2	0.088	0.003	2.957
Multiple	2	21.3	0.0	44.0	0.116	0.065	1.844

CI, confidence interval. SE, Standard error. p, two-sided *p*-value. Z, Z-statistic from the standard normal distribution.

### Prevalence of YFV infection in NHPs by immunohistochemistry

**Figure 5:**
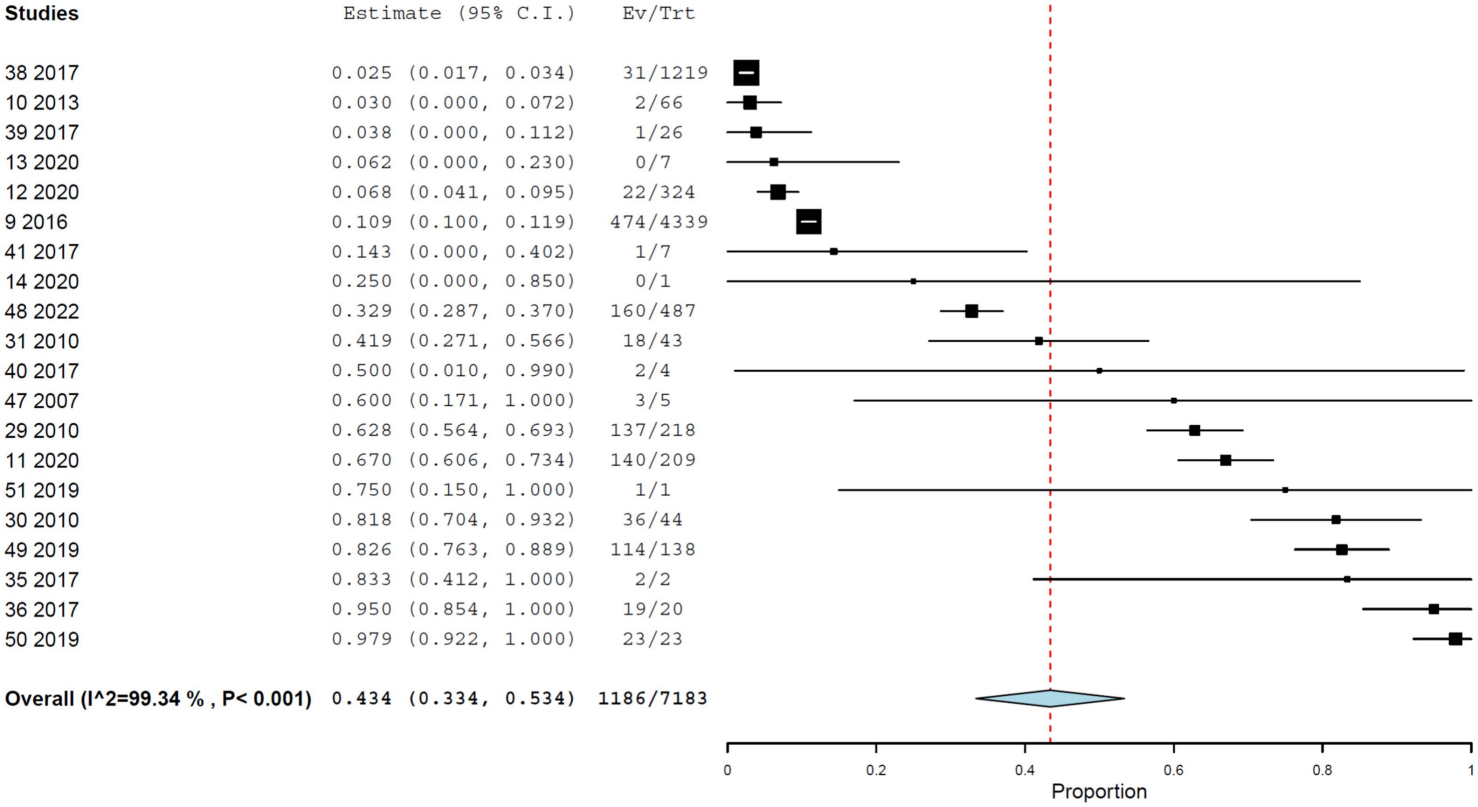
Prevalence of YF infection in NHPs by immunohistochemistry.

The pooled prevalence of YFV infection based on immunohistochemistry was 43.4% (95% CI, 33.4–53.4%), again with very high heterogeneity (I^2^ = 99.343%, τ^2^ = 0.042, Q^2^ = 2893.067) (Figure 5). *Allouatta* spp. accounted for the largest proportion of individuals assessed by immunohistochemistry (1,342, 18.6%) and showed a prevalence of 34.7% (95% CI, 10.6–58.8%).

### Seroprevalence of YFV infection in NHPs

#### Hemagglutination inhibition

**Figure 6:**
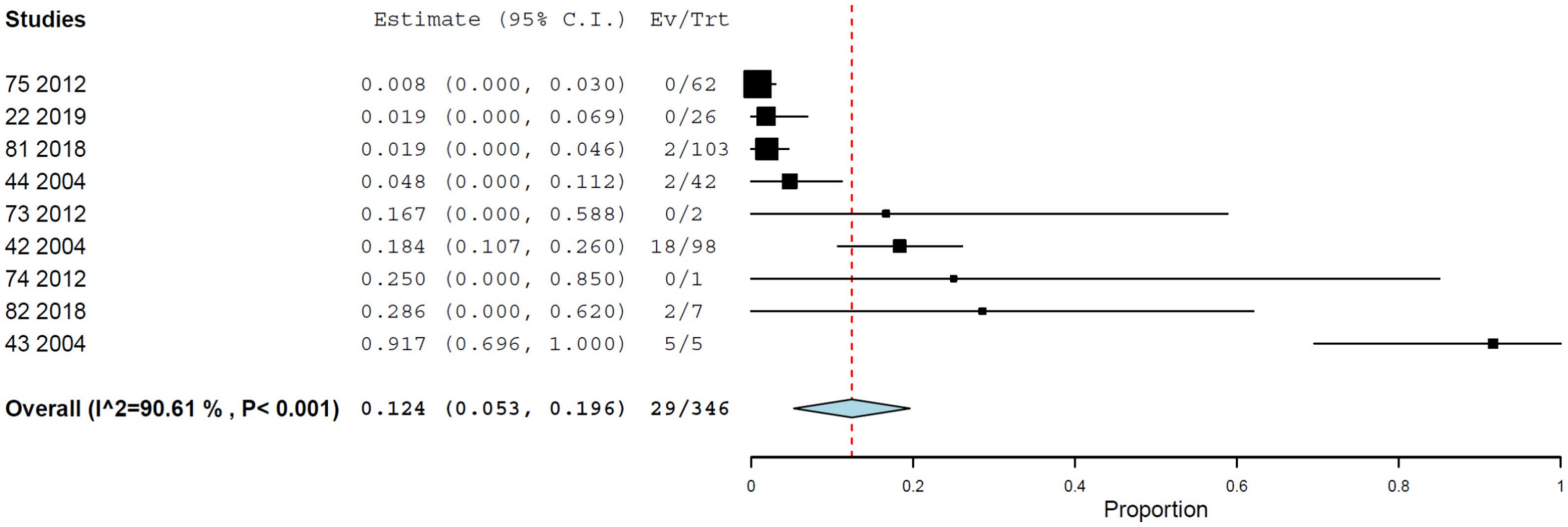
Seroprevalence of YF infection in NHPs by hemagglutination inhibition.

The pooled seroprevalence estimated by hemagglutination inhibition was 12.4% (95% CI, 5.3–19.6%), with substantial heterogeneity (*I*^2^ = 90.608%, τ^2^ = 0.007, *Q*^2^ = 85.180) (Figure 6). No significant differences were detected between NHP genera.

#### Plaque reduction neutralization test

**Figure 7:**
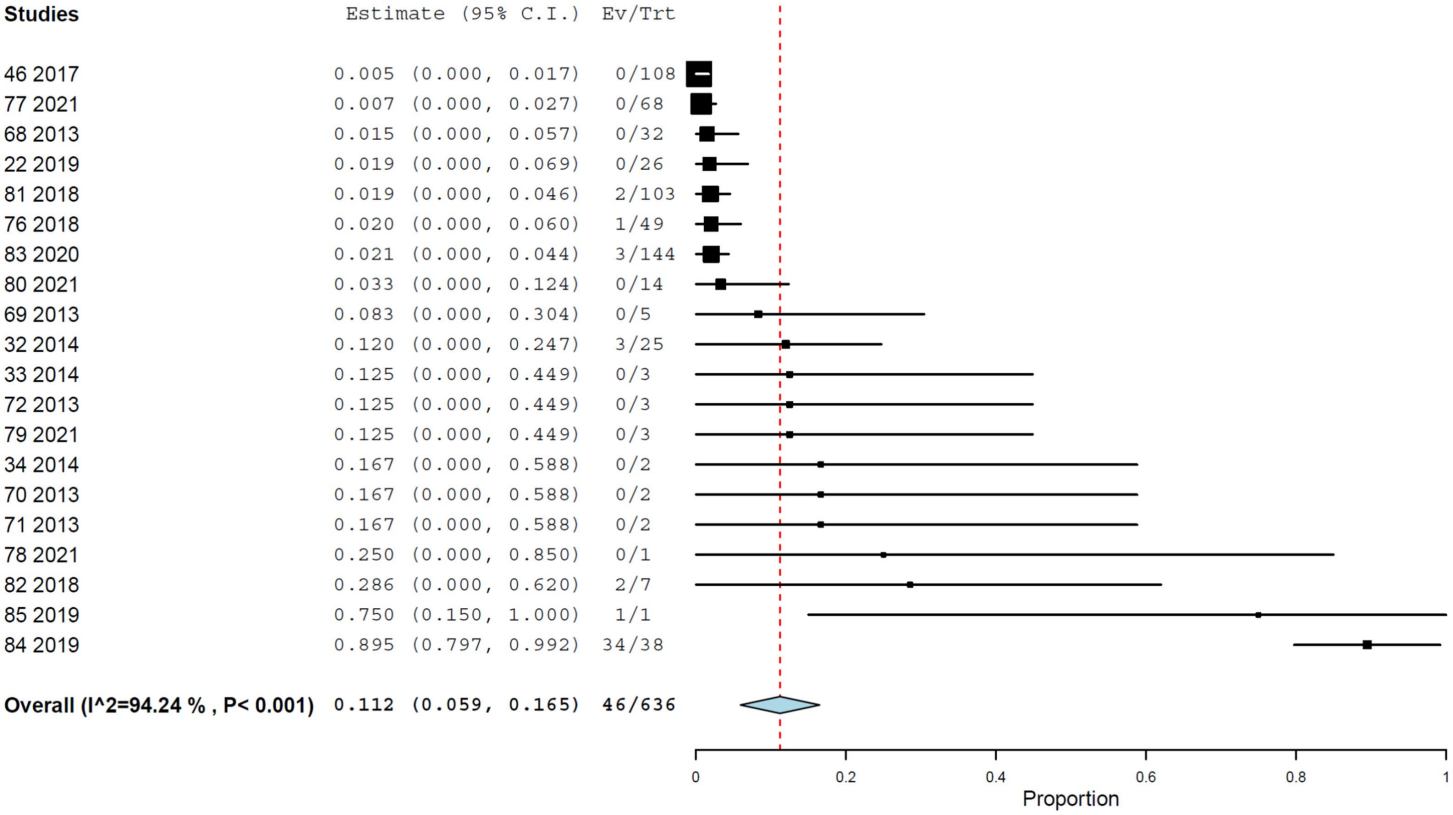
Seroprevalence of YF infection in NHPs by plaque reduction neutralization test.

**Figure 8:**
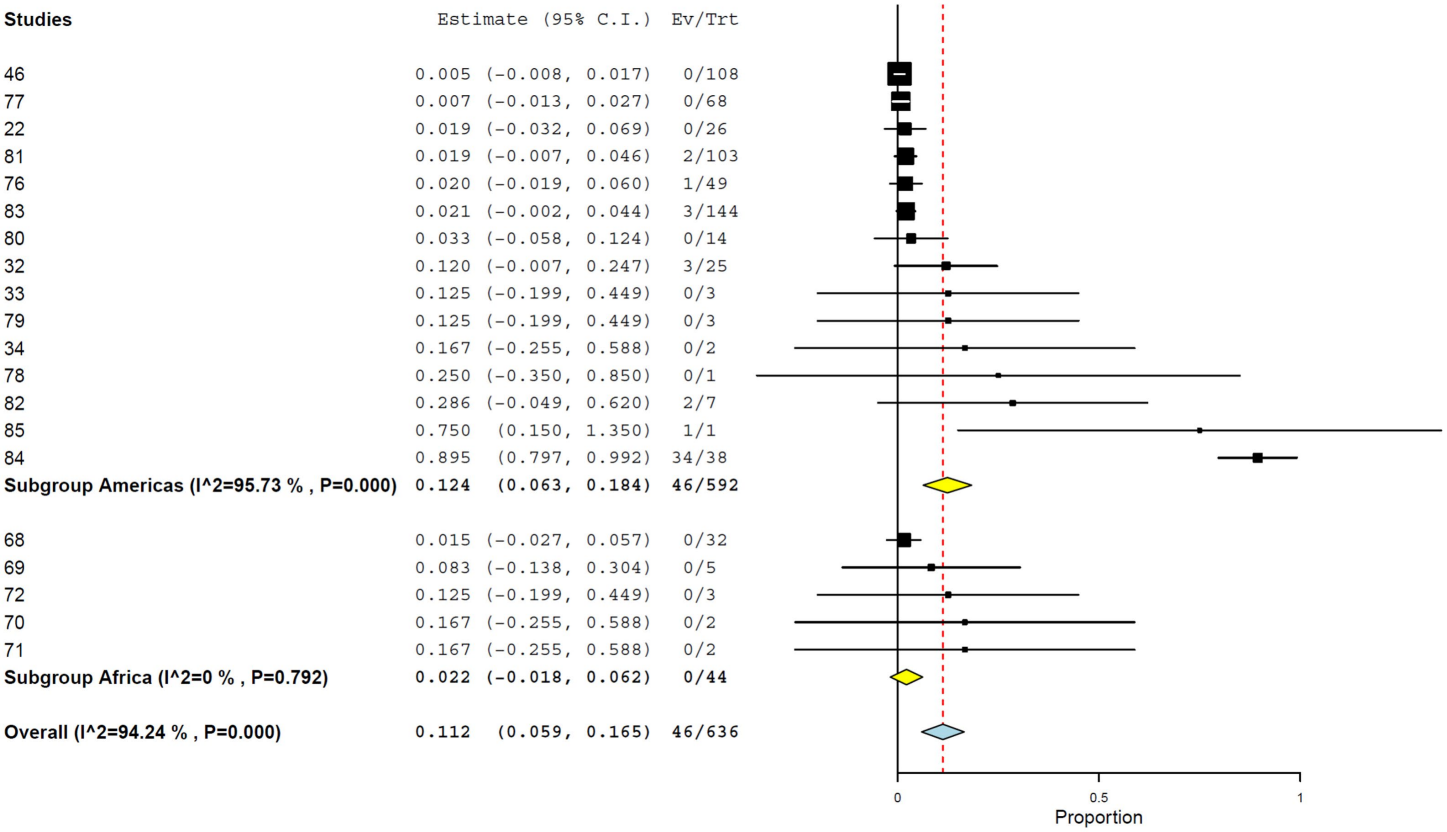
Seroprevalence of YF infection in NHPs by plaque reduction neutralization test by continents.

**Figure 9:**
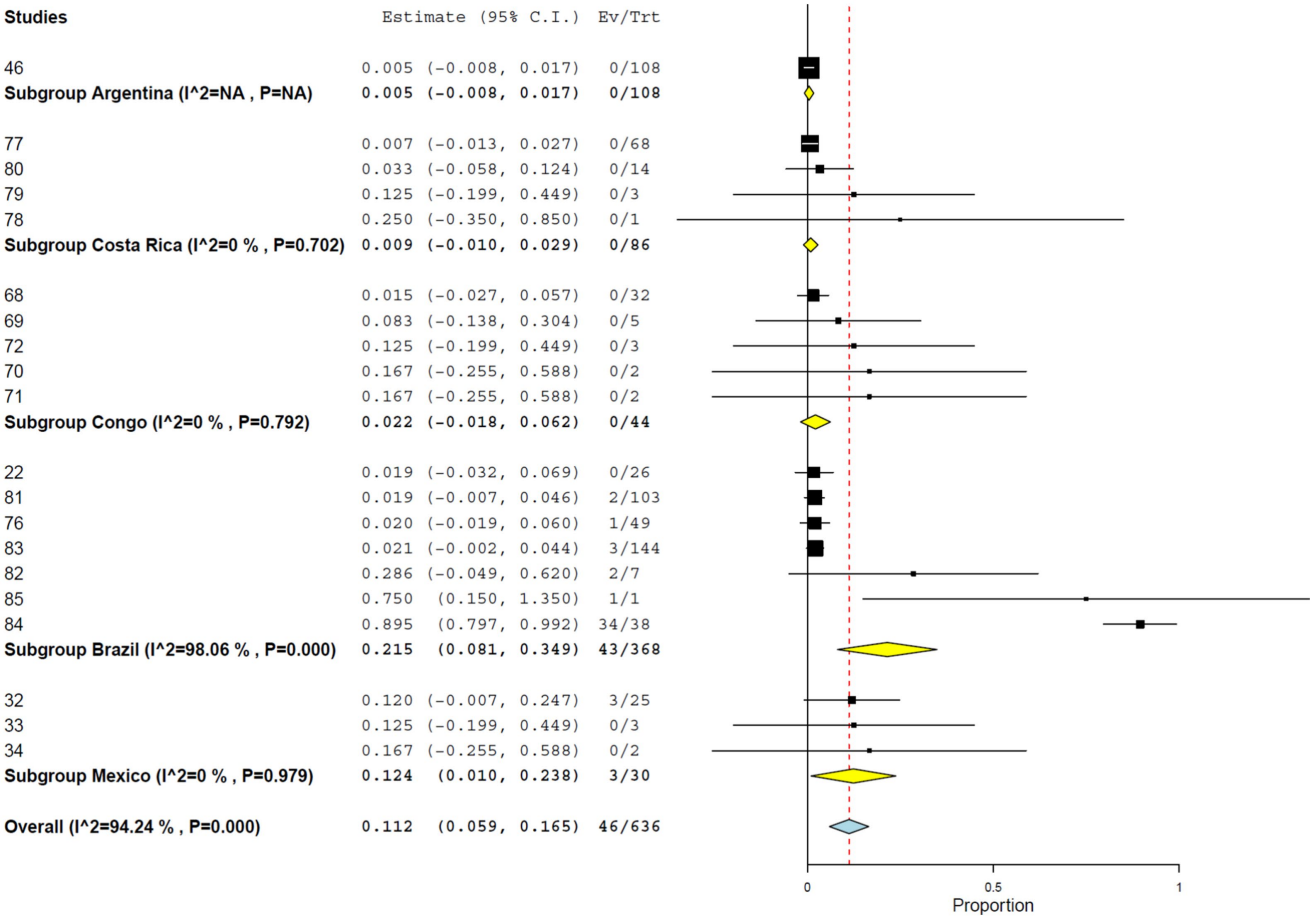
Seroprevalence of YF infection in NHPs by plaque reduction neutralization test by countries.

**Figure 10:**
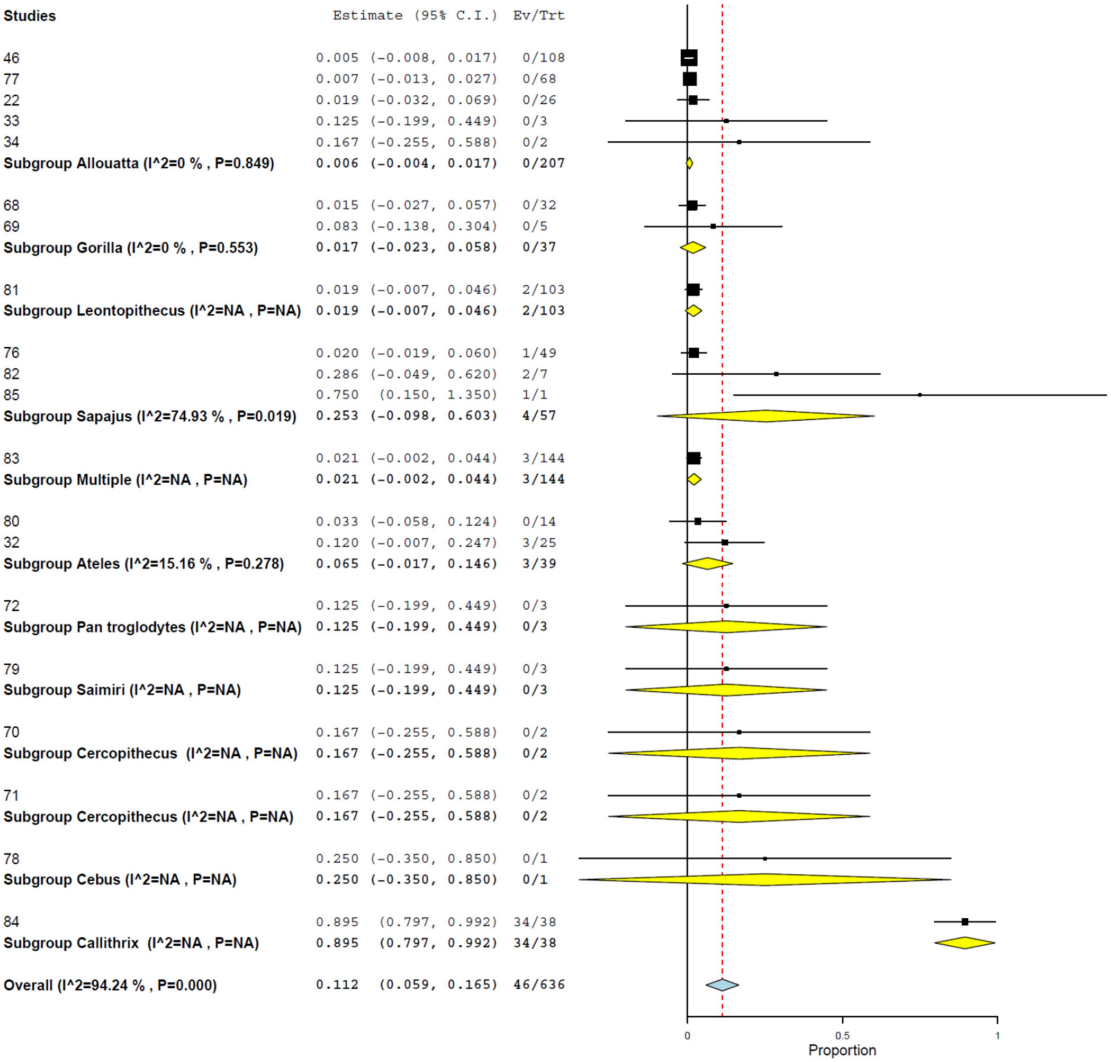
Seroprevalence of YF infection in NHPs by plaque reduction neutralization test by genus.

Using PRNT, the overall seroprevalence was 11.2% (95% CI, 5.9–16.5%), with high heterogeneity (*I*^2^ = 94.242%, τ^2^ = 0.008, *Q*^2^ = 329.959) (Figure 7). Seroprevalence was significantly higher in studies from the Americas (12.4%; 95% CI, 6.3–18.4%) than in those from Africa (2.2%; 95% CI, 0.0–6.2%) (Figure 8). At the country level, Brazil showed the highest seroprevalence (21.5%; 95% CI, 8.1–34.9%) (Figure 9). No significant differences in PRNT-based seroprevalence were observed across NHP genera (Figure 10).

#### Indirect immunofluorescence

Seroprevalence estimated by indirect immunofluorescence was 36.4% (95% CI, 0.0–74.9%), with very high heterogeneity (*I*^2^ = 98.615%, τ^2^ = 0.179, Q^2^ = 288.789), reflecting the limited number and diversity of available studies.

#### Mouse protection test

In NHP from Kenya assessed using the mouse protection test, seroprevalence was 5.0% (95% CI, 4.0–9.5%), with moderate to high heterogeneity (*I*^2^ = 65.586%, τ^2^ = 0.002, *Q*^2^ = 14.529).

#### Molecular, clinical, and pathological findings from individual case reports of YFV in NHPs

Ten publications reporting individual cases or small case series yielded 19 NHPs with confirmed YFV infection (Table 3). These cases were reported between 2003 and 2025, with a clear temporal concentration in recent years: almost two-thirds were published from 2020 onwards. Reporting increased particularly during 2023–2025, coinciding with intense epizootic activity in South America and including the first documented fatal cases in captive NHP in southern Colombia.

**Table 3:** Summary of case reports of YF in NHPs – Main features.

Title of publication	Year	Country	State or department	*Scientific name*	Sex	Alive/dead	RT-PCR	Samples	IHC	Samples	Genome sequencing	Lineage
First report of yellow fever virus in non-human primates in the State of Paraná, Brazil	2013	Brazil	Paraná	*Alouatta* sp.	Male	Dead			+	Liver		
Yellow fever surveillance challenge: Investigation of a marmoset non- autochthonous case	2020	Brazil		*Callithrix* spp.	Male	Dead	+	Liver	+	Liver, kidney, heart, lung, spleen, brain and cerebellum		
Phylogenetic analysis reveals a new introduction of Yellow Fever virus in São Paulo State, Brazil, 2023	2024	Brazil	São Paulo	*Callithrix* spp.		Dead	+	Liver	+	Liver, kidney and spleen	+	SA-I lineage 1E
	2024	Brazil	São Paulo	*Callicebus nigrifons*	Male	Dead	+	Liver	+	Liver, kidney and spleen		
Isolation and characterization of a Brazilian strain of yellow fever virus from an epizootic outbreak in 2009	2017	Brazil	Paraná	*Alouatta* spp.	Male	Dead	+	Liver, kidney, brain	+	Brain	+	YFV M17/09 (KX037022)
Coinfection with Canine Distemper Virus and Yellow Fever Virus in a Neotropical Primate in Brazil	2024	Brazil	Rio Grande do Norte	*Callithrix* spp.		Dead	+	Liver and spleen	+	Spleen		
Case report: Urbanized non-human primates as sentinels for human zoonotic diseases: a case of acute fatal toxoplasmosis in a free-ranging marmoset in coinfection with yellow fever virus	2023	Brazil	Brazilian	*Callithrix* spp.		Dead	+	Liver	+	Liver	+	YFV _PA/MG_ sub-lineage
Detection and Molecular Characterization of Yellow Fever Virus, 2017, Brazil	2018	Brazil	Minas Gerais	*Callicebus personatus*	Female	Dead	+	Liver, heart, spleen, lung, stomach and kidney			+	Genotype I
				*Callicebus personatus*	Female	Dead	+	Liver, heart, spleen, lung, stomach and kidney			+	Genotype I
Concurrent yellow fever and pulmonary aspergillosis due to *Aspergillus fumigatus* in a free-ranging howler monkey (Alouatta sp)	2020	Brazil		*Alouatta* spp.		Dead	+	Liver and lung	+	Liver and lung		
A case of yellow fever in a brown howler (*Alouatta fusca*) in Southern Brazil	2003	Brazil		*Alouatta fusca*	Female	Dead			+			
Fatal yellow fever among captive non-human primates in southern Colombia, 2025	2025	Colombia	Putumayo	*Cebus albifrons*	Male	Dead	+	Liver				
				*Ateles fusciceps*	Female	Dead	+	Liver				
				*Ateles fusciceps*	Female	Dead	+	Liver				
				*Ateles fusciceps*	Female	Dead	+	Liver				
				*Ateles fusciceps*	Female	Dead	+	Liver				
				*Lagothrix lagotricha*	Male	Dead	+	Liver				
				*Aotus* spp.	Male	Dead	+	Liver				
				*Aotus* spp.	Female	Dead	+	Liver				

##### Geographic and taxonomic distribution

All reported cases originated from South America, predominantly from Brazil (63%), followed by Colombia (37%). Brazilian reports were mainly from southern and southeastern states (Paraná, São Paulo, Minas Gerais, and Rio Grande do Norte), reflecting sustained sylvatic YFV circulation in these regions. Colombian cases were reported from Putumayo province, in the southern Amazon region, highlighting the expansion and persistence of YFV activity along this ecological corridor.

The affected animals represented at least six Neotropical genera, including howler monkeys (*Alouatta* spp.), marmosets (*Callithrix* spp.), titi monkeys (*Callicebus* spp.), spider monkeys (*Ateles fusciceps*), woolly monkeys (*Lagothrix lagotricha*), capuchins (*Cebus albifrons*), and night monkeys (*Aotus* spp.). Howler monkeys and marmosets accounted for nearly half of all cases, consistent with their well-established susceptibility to YFV infection and their role as key sentinel species in sylvatic transmission cycles.

##### Demographic characteristics and outcomes

Sex was reported in 12 of the 19 cases (seven males and five females); sex information was not available for the remaining animals. Age was rarely documented or explicitly reported, with only one study reporting it, precluding any meaningful age-stratified analysis. All animals were found dead or died shortly after the onset of illness, yielding a 100% fatal outcome among cases with documented clinical follow-up. This pattern should be interpreted cautiously, as case reports are inherently subject to reporting bias and tend to over-represent severe or fatal infections. Accordingly, the observed 100% fatality among reported cases should not be taken as an estimate of the true case-fatality rate of YFV infection in NHP populations. No survivors were documented, which nonetheless underscores the extreme susceptibility of many Neotropical primates to YFV infection and the rapid progression to death once overt clinical disease develops (Table 4).

**Table 4:** Summary of case reports of YF in NHPs – Clinical and necropsy findings.

Title of Publication	Case	Pyrexia	Emesis	Diarrhea	Runny nose	Vulvar discharge	Dehydration	Dyspnoea	Lethargy	jaundice	Hemorrhage	Anorexy	Paralysis	Splenomegaly	Hepatomegaly	Hemoperitoneum	Necropsy
First report of yellow fever virus in non-human primates in the State of Paraná, Brazil	1	nr	nr	nr	nr	nr	nr	nr	nr	+	nr	nr	nr	nr	nr	+	
Yellow fever surveillance challenge: Investigation of a marmoset non- autochthonous case	2	+	+	+	nr	nr	nr	nr	+	+	nr	nr	+	nr	+	nr	The liver showed mild periportal necrosis and rare apoptotic bodies, with minimal associated inflammatory infiltrate and moderate macrovesicular steatosis. The kidney showed chronic nephropathy, with marked tubular degeneration and dilation, interstitial inflammation and fibrosis, and membranous glomerulopathy. The spleen showed severe lymphoid depletion.
Phylogenetic analysis reveals a new introduction of Yellow Fever virus in São Paulo State, Brazil, 2023	3	nr	nr	nr	nr	nr	nr	nr	nr	nr	nr	nr	nr	nr	nr	nr	Absence of inflammation and hepatic steatosis.
	4	nr	nr	nr	nr	nr	nr	nr	nr	nr	nr	nr	nr	nr	nr	nr	The liver showed necrotizing hepatitis, with eosinophilic structures consistent with Councilman-Rocha Lima bodies, moderate steatosis, moderate ductular reaction, and a mild neutrophilic infiltrate. The spleen revealed marked depletion of the white pulp, with lymphoid necrosis and a neutrophilic infiltrate; the heart showed eosinophilic cardiomyocyte degeneration; and the kidney showed acute tubular necrosis.
Isolation and characterization of a Brazilian strain of yellow fever virus from an epizootic outbreak in 2009	5	nr	nr	nr	nr	nr	nr	nr	+	nr	+	nr	nr	nr	nr	nr	Yellow and congested liver.
Coinfection with Canine Distemper Virus and Yellow Fever Virus in a Neotropical Primate in Brazil	6	nr	nr	nr	nr	nr	nr	nr	nr	nr		nr	nr	nr	nr	nr	Hepatic necrosis, interstitial nephritis, and splenic lymphoid depletion of Malpighian corpuscles. Coinfection with Canine Distemper virus.
Case report: Urbanized non-human primates as sentinels for human zoonotic diseases: a case of acute fatal toxoplasmosis in a free-ranging marmoset in coinfection with yellow fever virus	7	nr	nr	nr	nr	nr	nr	nr	nr	nr	nr	nr	nr	+	+	nr	Multifocal random hepatocellular necrosis within scattered neutrophils and histiocytes. Coinfection with *Toxoplasma*.
Detection and Molecular Characterization of Yellow Fever Virus, 2017, Brazil	8	nr	nr	nr	nr	nr	nr	nr	nr	nr	nr	nr	nr	nr	nr	nr	Petechiae in the gastric mucosa, blood clots in the stomach contents, small yellowish areas in the hepatic parenchyma, edema and hyperemia of the eyelid, and mild pulmonary edema.
	9	nr	nr	nr	nr	nr	nr	nr	nr	nr	nr	nr	nr	nr	nr	nr	
Concurrent yellow fever and pulmonary aspergillosis due to *Aspergillus fumigatus* in a free-ranging howler monkey (*Alouatta* sp.)	10	nr	nr	nr	nr	nr	nr	nr	nr	nr	nr	nr	nr	nr	nr	nr	Mottled liver with a cinnamon-yellow color and splenomegaly. Microscopically, the liver showed severe diffuse hepatocellular necrosis and apoptotic bodies (“Councilman-Rocha-Lima bodies”). Concomitant angioinvasive pulmonary aspergillosis due to *Aspergillus fumigatus*.
A case of yellow fever in a brown howler (*Alouatta fusca*) in Southern Brazil	11	nr	nr	nr	nr	nr	nr	nr	nr	+	nr	nr	nr	nr	nr	nr	All mucous membranes, the intima of the large vessels, and the liver showed jaundice. The right kidney had an irregular area on its surface, and the spleen had uneven borders. Massive coagulative necrosis of most hepatocytes and fatty degeneration of the remaining cells were observed in the liver. Scattered apoptotic hepatocytes were observed. Renal tubular cells showed degenerative changes and hyaline casts in the lumen of numerous tubules. An extensive area of subacute interstitial nephritis in the right kidney corresponded to the irregular macroscopic area observed. Lymphoid follicles of the splenic white pulp showed variable central necrosis.
Fatal yellow fever among captive non-human primates in southern Colombia, 2025	12	+	nr	nr	nr	nr	nr	nr	+	+	nr	+	nr	nr	nr	nr	Interstitial and alveolar edema, hyaline membranes, congestion, pulmonary necrosis
	13	+	nr	nr	nr	nr	nr	nr	+	+	nr	nr	nr	nr	nr	nr	Generalized jaundice, cerebral hemorrhages, meningitis, pulmonary edema, cardiomegaly, pericardial effusion, hepatic necrosis, mesenteric lymphadenopathy
	14	nr	nr	nr	nr	nr	+	+	+	+	nr	nr	nr	nr	nr	nr	Rough coat, gallstones, severe intestinal parasitism (larvae and cysts), colonic gas, fermentation
	15	nr	nr	nr	nr	+	nr	nr	+	+	nr	nr	nr	nr	nr	nr	With gallstones, severe parasitism, gastritis, colonic gas, loss of muscle tone
	16	nr	nr	nr	nr	nr	nr	nr	nr	+	nr	nr	nr	nr	nr	nr	Interstitial and intra-alveolar edema, generalized hemorrhage, cardiomegaly, icteric pericardial effusion, myocarditis, icteric mesentery, intestinal larvae, meningoencephalitis
	17	nr	nr	nr	nr	nr	nr	nr	nr	+	nr	nr	nr	nr	nr	nr	Liver necrosis, jaundice, gastric ulcers, pancreatic necrosis, severe intestinal parasitism (with larval perforations), jaundiced intestinal mesentery, rough coat, cachexia
	18	nr	nr	nr	+	nr	nr	nr	nr	+	nr	nr	nr	nr	nr	nr	Pulmonary edema, myocarditis, hepatic necrosis, meningoencephalitis, and generalized hemorrhages
	19	nr	nr	nr	+	nr	nr	nr	nr	+	nr	nr	nr	nr	nr	nr	Pulmonary edema, myocarditis, hepatic necrosis, meningoencephalitis, and generalized hemorrhages

nr, not reported. +, positive.

##### Laboratory confirmation and molecular findings

YFV infection was confirmed primarily by RT-PCR, which was positive in 18 of 19 cases. Liver tissue was the most frequently tested specimen, although kidney, spleen, brain, heart, and lung samples were also used in selected cases. Immunohistochemistry was performed in 8 cases, revealing YFV antigen in hepatic tissue and, in several cases, in extrahepatic organs, including the spleen, kidney, lung, and brain (Table 4).

Genome sequencing or phylogenetic analysis was available for six cases. All sequences corresponded to South American genotype I strains, including SA-I lineage 1E and closely related sub-lineages, consistent with the genotypes known to be circulating during recent Brazilian and Colombian epizootics. These molecular findings support the regional persistence and spread of endemic YFV lineages, rather than repeated introductions of novel or exotic strains.

##### Clinical manifestations

Clinical information was available for approximately half of the reported animals (Table 4). When described, cases typically presented with acute systemic illness preceding death. Common manifestations included fever, marked lethargy, and jaundice, frequently accompanied by respiratory distress and anorexia. Neurological signs, including paralysis, meningitis, or meningoencephalitis, were documented in a subset of animals, indicating central nervous system involvement in severe infections. Hemorrhagic manifestations were less frequently documented but, when present, were associated with advanced multisystemic disease (Tables 5, 6).

**Table 5:** Summary of demographic, geographic, and laboratory features (*n* = 19).

Variable	n (%)
Country	
Brazil	12 (63%)
Colombia	7 (37%)
RT-PCR positive	18 (95%)
IHC performed	8 (42%)
Genome sequencing available	6 (32%)
Fatal outcome	19 (100%)
Coinfections reported	3 (16%)

**Table 6:** Most frequent clinical and pathological findings in the case reports.

Category	Finding	*n* (%)
Clinical	Jaundice	11 (58%)
	Lethargy	8 (42%)
	Pyrexia	6 (32%)
Pathology	Hepatic necrosis	14 (74%)
	Pulmonary edema/congestion	9 (47%)
	Hemorrhages	6 (32%)
	CNS involvement	4 (21%)

##### Necropsy and pathological findings

Necropsies were conducted in all 19 cases and revealed a broad spectrum of gross and microscopic lesions, with a strong predominance of hepatic pathology. Common findings included hepatic necrosis, necrotizing hepatitis, and Councilman-Rocha Lima bodies, often associated with diffuse or generalized jaundice. Pulmonary involvement was also prominent, with pulmonary edema, congestion, and interstitial or alveolar damage described in nearly half of the animals (Table 6).

Cardiovascular and neurological involvement were reported in several animals, including myocarditis, cardiomegaly, meningoencephalitis, and cerebral hemorrhages. Generalized or focal hemorrhages were observed in approximately one-third of animals, consistent with the hemorrhagic nature of severe YFV infection. Multisystemic involvement was common, with additional lesions affecting the kidneys, spleen, mesenteric lymph nodes, and gastrointestinal tract.

Coinfections were identified in three cases, including canine distemper virus, toxoplasmosis, and pulmonary aspergillosis. Although these concurrent infections may have contributed to disease severity, all affected animals ultimately succumbed to YFV infection, reinforcing the virus’s dominant pathogenic role in these cases.

Taken together, individual case reports of YFV infection in NHP depict uniformly fatal outcomes, extensive hepatic and multisystemic pathology, and infection with South American genotype I YFV strains. These observations reinforce the value of NHPs as highly sensitive sentinels for YFV circulation and highlight the importance of sustained epizootic surveillance to anticipate and mitigate the risk of human outbreaks.

## Discussion

Yellow fever remains a major public health and ecological challenge, despite the long-standing availability of an effective vaccine (20, 67). The present systematic review and meta-analysis, to our knowledge, provides the most comprehensive synthesis to date of YFV infection in NHPs, integrating more than seven decades of evidence from Africa and the Americas. By jointly analyzing prevalence, seroprevalence, and detailed epizootic case reports, this work offers a consolidated view of the role of NHPs in YFV transmission dynamics and identifies key gaps in surveillance, research, and prevention, gaps that are particularly relevant in the context of the ongoing resurgence of yellow fever in Latin America (68).

Our findings confirm that YFV infection in NHP is both widespread and highly heterogeneous, with substantial variation across regions, taxa, diagnostic methods, and time periods (16, 19, 21, 69, 70). Overall prevalence measured by molecular and histopathological methods was high, particularly in outbreak-driven investigations, reflecting the intense viral circulation that accompanies epizootic events. These results reinforce the concept that NHPs are not incidental hosts but central components of the sylvatic transmission cycle in the Americas. The consistently higher prevalence observed in genera such as howler monkeys and titi monkeys aligns with their recognized susceptibility and frequent involvement in large-scale die-offs. At the same time, the detection of infection across a broader range of Neotropical genera underscores that the YFV impacts a wider spectrum of primate species than is typically emphasized in surveillance and risk communication (45, 71, 72).

Seroprevalence estimates were generally lower than molecular prevalence, particularly in African studies, likely reflecting differences in transmission intensity, host susceptibility, post-infection survival, and study design. In the Americas, higher seroprevalence values suggest repeated or sustained exposure in some populations, whereas lower seroprevalence in Africa may indicate distinct ecological conditions, historical immunity patterns, or under-detection due to sparse sampling. Importantly, serological data complement molecular findings by documenting past exposure in apparently healthy animals, thereby capturing transmission that is invisible to mortality-based surveillance alone (58, 62).

One of the most striking features of the evidence base is its overwhelming concentration in the Americas, particularly in Brazil (18, 24, 31–33, 35–37, 40–42, 45–48, 51–53, 57, 58, 60, 62–64, 66). This geographic imbalance is notable given the long-standing endemicity of YF in Africa (43). The relative scarcity of African studies involving NHPs likely reflects logistical constraints, limited wildlife surveillance capacity, and a historical focus on human case detection. Consequently, the role of African primates in YFV ecology remains incompletely characterized, despite their importance in the virus’s evolutionary and historical context. This geographic imbalance limits the global generalizability of the pooled prevalence estimates presented here, which are largely driven by South American epizootic systems. Transmission dynamics, host susceptibility, and ecological drivers of YFV circulation may differ substantially between African and American ecosystems, which harbor distinct primate communities, vector species, and environmental conditions. Addressing these gaps will require expanded surveillance and research on NHPs in African endemic regions to provide a more balanced understanding of yellow fever ecology and to strengthen early warning systems for both animal and human outbreaks (73, 74).

The synthesis of individual case reports adds critical qualitative insights to the meta-analytic findings. The uniformly fatal outcomes observed among reported cases highlight the extreme susceptibility of many Neotropical primates to YFV and the rapid progression to death once clinical disease becomes apparent. Clinical and pathological patterns were remarkably consistent across genera, with predominant hepatic involvement, jaundice, pulmonary edema, hemorrhages, and, in some instances, central nervous system manifestations. These features closely mirror severe human yellow fever and underscore the value of NHPs as biological sentinels that reflect the pathogenic potential of circulating viral lineages (16, 58–66).

The inclusion of recent cases from captive and managed-care contexts further broadens the traditional view of YF epizootics (16, 19, 21). These reports demonstrate that captivity does not confer protection in endemic or high-risk environments and that wildlife rescue centers, conservation facilities, and peri-urban interfaces can experience severe outbreaks. The occurrence of fatal infections in threatened and endangered species has significant implications for biodiversity conservation and reveals an often-overlooked dimension of YF outbreaks (16, 19, 21). From a public health perspective, such events signal active viral circulation in areas where human exposure is plausible, highlighting the need for integrated surveillance that explicitly bridges wildlife, veterinary, and human health sectors (16, 19, 21).

As expected, heterogeneity was substantial across most meta-analyses, reflecting diverse study designs, diagnostic approaches, ecological contexts, and outbreak conditions. This high heterogeneity constrains the use of pooled estimates as precise measures of baseline endemic prevalence. However, it does not diminish their epidemiological value; rather, it underscores the dynamic, context-dependent nature of YFV transmission in NHP populations. Many included studies were undertaken in response to epizootic events, during which prevalence and mortality are intrinsically elevated. Non-probabilistic sampling, incomplete reporting of denominators, and limited characterization of study populations are common limitations that point to the need for more standardized, proactive surveillance frameworks (25, 75).

The temporal clustering of studies and cases is particularly relevant against the backdrop of the current yellow fever resurgence in South America (76). The concentration of publications after 2020 parallels intensified epizootic activity and increased awareness among surveillance systems (6). Recent data indicate ongoing YFV circulation in southeastern Brazil. For example, a phylogenetic analysis of epizootics detected between 2024 and 2025 in São Paulo State identified a lineage reintroduced from the Brazilian Midwest around 2022 that has persisted and spread locally, underscoring the importance of sustained NHP surveillance as an early warning system for human outbreaks (77). Recent analyses of long-term surveillance systems in Brazil further illustrate the value of systematic monitoring of non-human primate deaths. For example, a retrospective evaluation of yellow fever surveillance in the Federal District documented more than 1,100 epizootic events between 2008 and 2022, with laboratory-confirmed YFV infection in a small proportion of cases, and demonstrated the critical role of primate mortality surveillance as an early warning mechanism for human transmission (78).

In Venezuela, reports from 2025–2026 illustrate the magnitude of ongoing epizootic activity in the region, since more than 90 epizootic events in NHP were reported across eight Venezuelan states, accompanied by confirmed human cases and high case-fatality rates (79). Together, these findings point to the continued expansion of enzootic transmission corridors in northern South America and emphasize the urgent need for strengthened cross-border surveillance, vaccination strategies, and integrated One Health monitoring systems (77). Nevertheless, the largely reactive nature of current investigations indicates that opportunities for earlier detection and prevention are frequently missed. Systematic monitoring of both free-ranging and captive primate populations could improve the timeliness of public health interventions and reduce the risk of spillover into human communities (47).

From a One Health perspective, our findings illustrate the profound interconnectedness of ecological, social, and biological drivers of yellow fever transmission. Environmental change, including deforestation, habitat fragmentation, and agricultural expansion, reshapes vector habitats and increases contact patterns among mosquitoes, NHP, and humans. Climate variability further influences vector abundance and seasonality, while social factors such as occupational exposure, population mobility, informal settlements at forest edges, and inequitable access to vaccination intensify vulnerability. Within this complex system, NHPs occupy a critical position as amplifying hosts and early indicators of viral circulation (80, 81).

The absence of prior systematic reviews with meta-analysis focused specifically on YFV infection in NHPs represents a major gap that this study addresses (16, 19, 31, 42, 47, 51, 55). Previous reviews have predominantly emphasized human epidemiology, vector ecology, or outbreak narratives, with limited quantitative synthesis of animal data. By consolidating decades of fragmented evidence, our review provides a more robust platform for hypothesis generation, surveillance design, and policy formulation. The combined analysis of prevalence, seroprevalence, and case-based clinical-pathological data yields a more complete picture of infection dynamics than any single data stream alone.

Several key implications emerge. First, surveillance systems should formally integrate NHP data as a core component of yellow fever monitoring, rather than treating primate epizootics as ancillary observations (21). Second, diagnostic capacity for wildlife specimens must be strengthened, with standardized protocols and reporting practices that enable comparisons across regions and over time (16). Third, conservation considerations should be explicitly incorporated into outbreak response planning, particularly where endangered species are affected (19). Finally, human vaccination and vector control strategies should be informed by epizootic signals, especially in areas where primate mortality indicates active sylvatic transmission and elevated spillover (82).

## Limitations

This systematic review and meta-analysis have several limitations that should be considered when interpreting the findings. First, the quality of the primary studies was highly variable, and many were judged to be at high risk of bias. A large proportion of investigations were embedded in reactive, outbreak-focused surveillance systems that prioritize detecting infected or dead animals during epizootic events rather than in systematic wildlife probabilistic sampling. Such designs introduce selection bias and likely inflate prevalence estimates relative to baseline endemic transmission. Accordingly, pooled prevalence values are best interpreted as indicators of circulation intensity epizootic conditions, not as precise estimates of underlying endemic prevalence. Current surveillance strategies typically focus on detecting infected or deceased animals during epizootic events, rather than sampling representative populations, thereby limiting the generalizability of prevalence estimates. Furthermore, the predominance of outbreak-driven investigations may partially explain the high heterogeneity observed across the meta-analyses, as infection prevalence during epizootics is expected to be substantially higher than during inter-epizootic periods.

Second, the case-report synthesis is subject to pronounced reporting bias. The apparent 100% case fatality among reported NHP cases almost certainly reflects preferential publication of severe or fatal infections. Mild, subclinical, or self-limiting infections in NHPs are unlikely to be detected or reported, precluding estimation of a true case-fatality rate. In addition, incomplete reporting of denominators, study populations, sampling frames, and animal-level characteristics (e.g., age, sex, and habitat type) constrained our ability to conduct more refined subgroup analyses. A form of publication or reporting bias at the study level is also probable, as investigations documenting epizootic events or substantial primate mortality are more likely to be conducted and published than studies conducted during periods of low-level transmission. As a result, the available evidence base may disproportionately represent outbreak settings, potentially contributing to overestimation of prevalence in pooled analyses.

Third, geographic and taxonomic coverage was uneven. Most data originated from the Americas, especially Brazil, whereas large regions of Africa and other potential endemic or at-risk regions were underrepresented or absent. This imbalance limits the extrapolation of our findings to understudied regions and hinders formal comparative analyses across continents. Moreover, the literature is heavily skewed toward a subset of highly susceptible genera, leaving important gaps regarding many other primate species.

Diagnostic heterogeneity represents an additional limitation. Included studies employed a wide array of laboratory methods, each with distinct sensitivity, specificity, and temporal windows of detection. RT-PCR is highly sensitive during acute infection and is widely considered the reference standard for confirming YFV infection in both humans and NHPs, whereas immunohistochemistry is particularly valuable for post-mortem confirmation and spatial localization of viral antigen in tissues. Serological assays, including hemagglutination inhibition and plaque reduction neutralization tests, primarily inform past exposure and may underestimate recent infections or be affected by cross-reactivity with other flaviviruses. Emerging molecular approaches such as reverse transcription loop-mediated isothermal amplification (RT-LAMP) offer promising, rapid, field-adaptable alternatives for wildlife surveillance; for example, Cardoso et al. (83) developed and validated an RT-LAMP assay for YFV detection in NHP samples from southern Brazil, demonstrating its potential for decentralized surveillance settings. Future surveillance efforts would benefit from harmonized diagnostic algorithms that integrate molecular, histopathological, and serological approaches to improve comparability across studies and regions. It is also likely that reliance on published studies led to underestimation of the true burden of YFV infection in NHPs, as many epizootic events, particularly in remote or resource-limited settings, may go undetected or unreported. Together, these limitations highlight the need for standardized surveillance protocols, improved reporting practices, expanded geographic coverage, and stronger international collaboration to generate more robust and comparable data on yellow fever virus infection in non-human primates. Finally, although we attempted to register the review protocol, we encountered technical and scope-related obstacles in PROSPERO, including the lack of suitable registration options for systematic reviews of observational wildlife and outbreak studies. The absence of formal protocol registration is therefore acknowledged as an additional methodological limitation.

## Conclusion

This systematic review and meta-analysis demonstrate that YFV infection in NHPs is widespread, often severe, and epidemiologically pivotal across endemic regions, particularly in the Americas. By synthesizing more than seven decades of data on prevalence, seroprevalence, and epizootic case reports, we provide the most comprehensive assessment to date of YFV circulation in NHPs. The findings reinforce the central role of NHPs as highly sensitive sentinels of YF activity and underscore their importance in detecting viral circulation prior to or concurrent with human outbreaks. Recent genomic surveillance in São Paulo State illustrates that systematic monitoring of epizootic events in Neotropical primates can detect newly introduced viral lineages and help guide public health responses in areas at risk of human transmission (77). The consistently high infection prevalence observed during epizootic periods, coupled with the uniformly fatal outcomes in reported cases, underscores both the vulnerability of many primate species and the intensity of transmission during active outbreaks.

In the context of the ongoing resurgence of yellow fever in Latin America, the evidence synthesized here has direct implications for surveillance, prevention, and response. Detection of infection across multiple primate genera, in both free-ranging and captive contexts, supports expanding epizootic surveillance beyond traditional sentinel species and sites. Integrating NHPs monitoring into routine public health systems can enhance early warning capacity, inform targeted vaccination campaigns, and support timely vector control interventions. From a One Health standpoint, strengthening the integration of animal, human, and environmental data will be essential to mitigate future outbreaks, safeguard vulnerable wildlife populations, and reduce the risk of spillover to humans. Collectively, this review establishes a critical evidence base to support more proactive, integrated, and sustainable approaches to yellow fever control in an era of accelerating ecological change and renewed epidemic risk.

## Data Availability

The original contributions presented in the study are included in the article/Supplementary material, further inquiries can be directed to the corresponding author/s.
