# Supplementary material for: Yellow fever virus infection in non-human primates: a systematic review and meta-analysis of prevalence, seroprevalence, and epizootic case reports (1950–2025)
Source: Front Vet Sci. 2026 Mar 31;13:1798623. doi: 10.3389/fvets.2026.1798623 (PMC13080213; doi:10.3389/fvets.2026.1798623)
Supplement: Supplementary file 1 [file Supplementary_file_1.docx]

# Supplementary Materials

# Table S1. Search Strategy Used for the Systematic Review.

**Databases Searched**

Scopus; PubMed; Web of Science; SciELO

**Search Period**

From database inception (1950) to December 1, 2025

**Language Restrictions**

None

**Study Design Filters**

Observational studies (prevalence or seroprevalence). Case reports were included for qualitative synthesis only.

**PubMed Search Strategy**

(("Yellow Fever"[Mesh] OR "yellow fever virus" OR "yellow fever")
AND
("Primates"[Mesh] OR "non-human primates" OR "nonhuman primates" OR monkey* OR primate*))

Filters applied: none

**Scopus Search Strategy**

(TITLE-ABS-KEY("yellow fever" OR "yellow fever virus")
AND
TITLE-ABS-KEY("non-human primate*" OR "nonhuman primate*" OR primate* OR monkey*))

**Web of Science Core Collection Search Strategy**

TS=("yellow fever" OR "yellow fever virus")
AND
TS=("non-human primate*" OR "nonhuman primate*" OR primate* OR monkey*)

Indexes searched: SCI-EXPANDED, SSCI, ESCI

**SciELO Search Strategy**

("yellow fever" OR "yellow fever virus")
AND
("non-human primate*" OR "nonhuman primate*" OR primate* OR monkey*)

**Additional Notes**

- No filters for geography, species, or diagnostic method were applied.
- Reference lists of included articles were screened manually to identify additional relevant studies.
- Case reports and case series were retained for a separate qualitative analysis of molecular, clinical, and pathological findings.

# Table S2. Risk of Bias Assessment Using the Joanna Briggs Institute Tool.

Risk of bias was assessed using the Joanna Briggs Institute (JBI) Critical Appraisal Checklist for Prevalence Studies. Judgements were made at the study level.

| **Study (short title)** | **Q1 Sampling** | **Q2 Population described** | **Q3 Valid measurement** | **Q4 Standard measurement** | **Q5 Analysis** | **Overall risk** |
| --- | --- | --- | --- | --- | --- | --- |
| YF reemerging in São Paulo | No | Yes | Yes | Yes | No | High |
| YF epizootics in NHPs | No | Yes | Yes | Yes | No | High |
| Histopathology in howlers | No | Yes | Yes | Yes | No | High |
| YF epizootics Brazil | No | Yes | Yes | Yes | No | High |
| Non-sylvatic transmission | No | Yes | Yes | Yes | No | High |
| Persistent YFV circulation | No | Yes | Yes | Yes | No | High |
| Ecological drivers enzootic YF | No | Yes | Yes | Yes | No | High |
| Flavivirus antibodies | No | Yes | No | No | No | High |
| YF protection tests review | No | Unclear | No | No | No | High |
| YF outbreak in Allouatta | No | Yes | Yes | Yes | No | High |
| YF in titi monkeys | No | Yes | Yes | Yes | No | High |
| Serological evidence YF | No | Yes | No | No | No | High |
| YF outbreak NHPs | No | Yes | Yes | Yes | No | High |
| Histopathologic susceptibility | No | Yes | Yes | Yes | No | High |
| Wild mammals and YF | No | Unclear | No | No | No | High |
